# Navel Healing and Calf Fitness for Transport

**DOI:** 10.3390/ani12030358

**Published:** 2022-02-01

**Authors:** Mariana Roccaro, Marilena Bolcato, Naod Thomas Masebo, Arcangelo Gentile, Angelo Peli

**Affiliations:** 1 Department for Life Quality Studies, Alma Mater Studiorum University of Bologna, Corso D’Augusto, 237 Rimini, Italy; mariana.roccaro2@unibo.it (M.R.); angelo.peli@unibo.it (A.P.); 2 Department of Veterinary Medical Sciences, Alma Mater Studiorum University of Bologna, Via Tolara di Sopra, 50, Ozzano dell’Emilia, 40064 Bologna, Italy; naodthomas.masebo2@unibo.it (N.T.M.); arcangelo.gentile@unibo.it (A.G.)

**Keywords:** calf, navel healing, umbilicus, transport, health, welfare, law

## Abstract

**Simple Summary:**

In the dairy industry, for male calves, the costing and balancing of animal welfare and farmers’ interests when determining the optimum age for a calf to leave the farm of origin is a challenge. In the European Union, calves whose navel has not “completely healed” cannot be transported. This study aimed to clarify what is meant by “navel healing”, as no specific definition is provided by the law, giving raise to different interpretations. The navels of 299 dairy calves (55 males, 244 females) aged 0–90 days were examined and scored. Our results show that a completely dry and shriveled navel stump entails a high risk of transporting too young calves, whilst the presence of a scab covering the umbilical wound could be considered acceptable for short journeys, as the risk of transporting calves that are too young is low. “Navel healing” should be defined as the scarring of the umbilical wound, which occurs no earlier than 3–4 weeks of life. Transporting calves with a completely healed navel should be considered best practice because it ensures that calves that are too young are not transported and therefore guarantees higher animal welfare standards.

**Abstract:**

Dairy male calves are at risk of welfare compromise as they are usually transported at a very young age. The European Union has set a “completely healed navel” requirement for calf transport; moreover, a minimum age is established for longer journeys. However, this requirement has proven to be prone to misinterpretation. This study aimed to clarify what is meant by “navel healing” and to provide strong elements for reaching a consensus. The navels of 299 dairy calves (55 males, 244 females) aged 0–90 days were examined and scored 1 to 5 according to their healing status. Based on our results, a completely dry and shriveled navel (score 3) would imply a 25.5–38.0% risk of transporting too young calves. Alternatively, the presence of a scab covering the umbilical wound (score 4) would entail a 4.3% risk of transporting calves less than 10 days old and could be considered good practice for transporting calves (except for journeys exceeding 8 h). Conversely, complete navel healing (score 5) guarantees that calves that are too young are not transported; therefore, it should be considered best practice for transporting calves in general and the minimum requirement for transporting calves for journeys exceeding 8 h.

## 1. Introduction

Live animal transport poses a risk to animal health and welfare and this risk is particularly relevant when the transported animals are young. The higher sensitivity of young animals, such as calves, to transport stress is due, on the one hand, to the immaturity of their immune system, the incomplete development of the hypothalamic–pituitary axis, and their reduced ability to thermoregulate and, on the other hand, to exposure to a multitude of novel stressful stimuli, such as handling, loading, weighing, regrouping with unfamiliar animals, deprivation of food and water, and fluctuating temperatures [1,2,3,4].

Up-to-date statistics on calf transport mortality rates are limited. In Australia, an average mortality rate of 0.64% has been reported for bobby calves (less than one week old) over the period 1998–2000 [5]. Later surveys carried out by the New Zealand Government reported a decrease in mortality rates from 0.68% in 2008, to 0.25% in 2015, and 0.12% in 2016, following farmer education campaigns and the implementation of voluntary initiatives [6]. A study documenting animal transits through the Southern Italian control post from 2010 to 2015 reported a mortality rate of 0.011% for medium-sized calves (<100 kg) and of 0.044% for heavy calves (200 kg), but no information was given regarding the age of the examined animals [7].

Even though few calves die during transport, a strong negative correlation has been shown between age at transport and mortality, which occurs within few weeks after transport due to secondary infections resulting from the impairment of immune system function caused by transport stress [8].

Dairy calves, especially males, are at particular risk of welfare compromise as they are usually transported at a very young age to slaughterhouses (e.g., in Australia and New Zealand) or to white veal production facilities (e.g., in European countries, including Italy) and they often show impaired health conditions upon arrival. Indeed, male gender has been identified as a significant risk factor for increased mortality and unwanted early slaughter in veal production facilities [9], presumably because the nursing care of dairy male calves might be neglected due to their low economic value [10,11].

The correlation between failure of passive transfer of colostral immunoglobulins, morbidity, and mortality is well documented [12,13]. However, studies on the relationship between good passive immunity transfer and infectious disease risk in calves have generated conflicting results regarding umbilical infections, thus suggesting that other management and environmental factors are also important [14,15].

Navel inflammation is one of the most common health problems reported upon arrival at white veal facilities or auction sites, with a prevalence ranging from 20% to 32% [16,17,18,19], and it has been associated with an increased risk of mortality in the first three weeks after transport [20]. Umbilical infections are among the main causes for neonatal calf mortality and carcass condemnation, as they often evolve into septicemia with endocarditis, arthritis, hepatitis, meningitis, and, eventually, death [21,22].

Calf age and navel condition are commonly used as indicators for fitness in several regulations for the protection of transported animals. In the European Union, according to the Council Regulation (EC) No 1/2005, calves cannot be transported if their navel “has not completely healed” (Annex I, chapter I). Moreover, “calves of less than 10 days of age” cannot be transported for more than 100 km (Annex I, chapter I) and for journeys that exceed 8 h they must be “older than 14 days” (Annex I, chapter VI) [23]. As another example, according to the Australian and New Zealand regulations, calves can be transported if they are at least 4 full days (96 h) old and their navel cord is wrinkled, withered, and shriveled [24,25]. In Canada, calves that are 8 days of age or less can be transported only once and must not have an unhealed or infected navel [26].

The scientific literature concerning the post-natal evolution of umbilical structures is limited. At birth, the umbilical stump is red-pink, flexible and hydrated. It then undergoes a drying and mummification process; at 5 days of age it turns brown-black and becomes inflexible and shriveled. At about 14 days of age it falls off, leaving a wound that is soon covered by a scab. Around 3–4 weeks of age, the umbilical wound is completely healed [27,28].

Since the healing process of the umbilical wound seems to extend well beyond 10 days of age, what the European legislator means by the term “healed” seems unclear. Contradictory terminology has also been found in the OIE Terrestrial Animal Health Code, where article 7.3.7 states that “animals that are unfit to travel include newborns with an unhealed navel”, while article 7.11.7 says that “recently born calves should not be transported until the navel is dry” [29].

The aim of the present study is to contribute to clarify what is meant by “navel healing”, since no specific definition is provided by the law, giving raise to different interpretations. The ultimate aim is to provide strong elements for reaching a consensus among farmers and veterinary practitioners in order to comply with the European regulation. In order to do so, a review of the available literature on navel healing and the direct examination of calves in the first weeks of life were performed. A scoring system to help to avoid misinterpretation of the healing status of the navel is presented.

## 2. Materials and Methods

A total of 299 dairy calves (55 males, 244 females) aged 0–90 days were included in this study. The calves were mainly Holstein breed (n = 201) and crossbreds (n = 98) reared in 5 dairy cattle farms located in Bologna and Modena provinces (Emilia-Romagna region). Calves were housed in ground-level individual igloos (4 farms) or elevated cages (1 farm) with straw bedding up to 2 months of age and then placed in group pens. The hygiene level of the pens and straw bedding was between good and excellent. The pens were located outdoors in a sheltered area, protected from adverse climatic conditions. All calves were identified with two ear tags bearing the calf’s identification number and the date of birth. Only data obtained from farms where birth registration was reliable were used. In all farms it was standard practice to treat the navel stump of both male and female calves with a tetracycline-based spray at least once at birth. However, it was not always possible to verify whether this was done. It was also not possible to collect individual information on colostrum administration.

**Figure 1:**
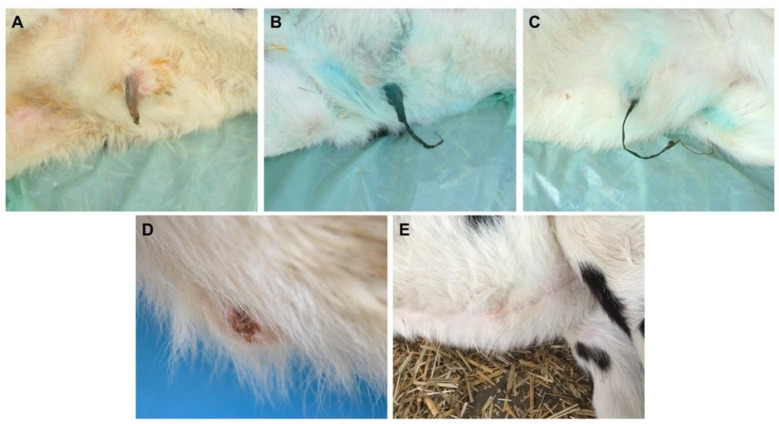
Healing status of the umbilical wound and corresponding score: (**A**) red-pink color, hydrated, flexible (score 1); (**B**) crimson-purple color, flattened, dry in its distal portion (score 2); (**C**) brown-black color, completely dry and shriveled, inflexible (score 3); (**D**) no umbilical stump, but scab on the umbilical wound (score 4); (**E**) completely healed umbilical wound (score 5).

The calves’ umbilical stumps were inspected and palpated on a single occasion between January and March 2021. Based on the post-natal evolution of the umbilical structures described in the literature [27,28], a score corresponding to the different stages of umbilical healing was attributed as follows: (1) red-pink color, hydrated, flexible; (2) crimson-purple color, flattened, dry in its distal portion; (3) brown-black color, completely dry and shriveled, inflexible; (4) no umbilical stump, but scab or granulation tissue on the umbilical wound; (5) completely healed umbilical wound (Figure 1). The inspections were carried out by three observers (M.B., A.G., M.R.) with experience in bovine medicine. The first visit was carried out together and the score was agreed upon. Calves showing signs of omphalitis (e.g., enlarged umbilicus, pain upon palpation, draining of purulent material) were excluded from the study.

Statistical analysis was performed using Microsoft Excel (v16.16.10, Microsoft, 2018) and XLSTAT (v2016.5, Addinsoft, 2016).

The correlation between calf age and umbilical score was investigated by calculating the Spearman’s rank correlation coefficient (the significance level was set at 0.05).

The two variables “calf age” and “umbilical score” were then converted into binary variables and square contingency tables were generated analyzing different scenarios. Regarding calf age, the threshold was either set at 10 days (journeys longer than 100 km), or 14 days (journeys exceeding 8 h), as clearly established by law. Regarding umbilical score, given the lack of clarity on what the European legislator means by “healed navel”, three possible cases were considered: in the first case, the threshold was set at score 3 (completely dry and shriveled, inflexible umbilical stump); in the second case, the threshold was set at score 4 (no umbilical stump, but scab or granulation tissue on the umbilical wound); in the third case, the threshold was set at score 5 (completely healed umbilical wound). The probability of finding the relevant observed values was calculated.

## 3. Results

The age distribution of the calves included in our sample was as follows: 0–9 days (n = 94), 10–14 days (n = 35), 15–20 days (n = 43), 21–30 days (n = 51), 31–40 days (n = 29), 41–50 days (n = 24), and 51–90 days (n = 23).

The age distribution of calves with different umbilical scores is shown in Table 1.

The umbilical score was moderately correlated with calf age (R= 0.604; *p* < 0.0001).

All the calves with umbilical score 1 were less than 10 days old. The majority of calves with score 2 were less than 10 days of age; however, the navel stump was dry only in its distal portion also in a 10-day-old calf and in two 15-day-old calves. The 47% of the calves with a completely dry and shriveled navel stump (score 3) were aged less than 10 days, but the navel stump was still present even in calves over one month old (6%). Conversely, calves with score 4 (no umbilical stump, but scab or granulation tissue on the umbilical wound) were distributed over all the observed age ranges, from 1 day to 2 months old. Finally, in our sample, only two calves less than 3 weeks old (specifically, 19 and 20 days of age) had a completely healed navel, i.e., only 3% of all calves with score 5.

The contingency tables created for the analysis of the different scenarios are shown in Table S1, Table S2, Table S3, Table S4, Table S5 and Table S6; the results are summarized in Table 2, which shows the probability that calves with an umbilical score of at least 3 (completely dry and shriveled, inflexible umbilical stump), 4 (no umbilical stump, but scab or granulation tissue on the umbilical wound), or 5 (completely healed umbilical wound) were less than 10 days old (fit for journeys within 100 km), at least 10 days old (fit for journeys longer than 100 km), or older than 14 days (fit for journeys exceeding 8 h).

## 4. Discussion

Calf age is a critical factor affecting transport-related stress, and a strong negative correlation exists between age at transport and mortality [8]. Nevertheless, there is no scientific consensus on the optimal age for transporting calves [30].

Navel inflammation is among the most common diseases and causes of mortality upon arrival to white veal facilities of shortly after in transported calves, with a prevalence of up to 32% [14,16,17,21]. Furthermore, the pain associated with navel inflammation has been identified as a factor reducing calves’ lying time during the first 2 weeks after transport, thus interfering with disease recovery [31].

The legislator’s response to these challenges has been to develop and implement regulations that limit calf age and health status as well as transport duration and relate to conditions. For example, the European Union has set a “completely healed navel” for calves to be deemed fit for transport; moreover, a minimum age of 10 or 14 days is required for journeys that exceed 100 km or 8 h, respectively.

Apart from a few exceptions [27,28], research on the most authoritative textbooks of veterinary internal medicine, clinical pathology, surgical pathology, and obstetrics failed to provide information on the healing times of the umbilical wound [32,33,34,35,36].

On the basis of the available literature, the navel stump falls at 14 days of age on average. This event is followed by the formation of a firm, thick scab, which takes a few days to occur and precedes healing. As the various umbilical structures heal at different times, it is necessary to ensure that the external umbilicus (i.e., the main entry point for pathogens and the only structure that can be assessed on inspection) is completely healed. The physiological endpoint of mammalian wound repair displays the formation of a scar [37]. Therefore, so-called “navel healing” should be defined as the scarring of the umbilical wound (score 5), which occurs no earlier than 3–4 weeks of life.

The results of our study corroborate this concept. In our sample, 90% of all calves up to 14 days old still had the navel stump, which in most cases was completely dry and shriveled (score 3). Most of the calves with score 4 (no umbilical stump, but scab or granulation tissue on the umbilical wound) were between 15 and 40 days old. However, 18% of calves with score 4 were between 1 and 14 days of age; it was not possible to determine whether this was due to early navel stump detachment or whether it resulted from a rupture of the navel stump at the external umbilicus at birth. The youngest calves with a completely healed navel (score 5) were 19 and 20 days old, whilst the highest percentage of calves with score 5 (31%) was between 21 and 30 days old. We cannot exclude that the navels of the calves over 1-month-old had healed before that age; however, it is highly unlikely that a calf will have a completely healed navel (score 5) before 3 weeks of age.

Our data support the notion that only a completely healed navel (score 5) would prevent calves from being transported too young. In fact, according to our findings, if the minimum requirement were a completely dried and shriveled navel (score 3), there would be a 25.5% risk of transporting calves less than 10 days old for journeys over 100 km, and a 38% risk of transporting calves younger than 14 days old for journeys exceeding 8 h. Alternatively, a score of 4 (no umbilical stump, but scab or granulation tissue on the umbilical wound) as a minimum requirement would still entail a risk of transporting calves that are too young, albeit much lower (i.e., 4.3% risk of transporting calves less than 10 days old for journeys over 100 km and 8.7% risk of transporting calves younger than 14 days old for journeys exceeding 8 h). Conversely, with score 5 it would be reasonably certain that calves are at least 10 days old or older than 14 days.

Consequently, both in score 3 and score 4 scenarios, other elements would be necessary to verify whether the age of the calf complies with the legal requirements concerning transport duration. How, though, can one be sure of the calf’s age? Calf age cannot be reliably determined from physiological or physical characteristics. A recent study investigating the accuracy of serum gamma glutamyl transferase and body weight as predictors of age in young calves found only a moderate correlation [38]. Given this limitation, the only element to determine it is documentation. In the European Union, according to Regulation (EC) No 1760/2000 and, since 21 April 2021, the Commission Implementing Regulation (EU) 2021/520 laying down rules for the application of Regulation (EU) 2016/429, with regard to the traceability of certain kept terrestrial animals, bovine animals shall be identified by a physical means of identification approved by the competent authority (e.g., ear tag) bearing a unique identification code, which makes it possible to identify each animal individually along with the holding on which it was born. The means of identification may be applied and registered by the farmer on the national database within a period that shall not exceed 20 days from the date of birth and in any case before the animals are moved from the farm. As a result of this time allowance, it is possible that some calves are falsely registered as having an older age so that they can be transported earlier. Against this background, the element on which competent authorities base their judgement in determining calf fitness for transport is navel status.

The navel healing process may also be affected by different navel care practices. In a study investigating the efficacy of light-emitting diode (LED) phototherapy on navel healing in 57 newborn calves, the umbilical stump of all animals fell off by the 25th day of age; on average, it fell off 3 days earlier in LED-treated calves (*p* < 0.01). At 30 days of age, the umbilical wound had healed in almost all the LED-treated calves (96.4%) but only in 69% of the calves in the control group [39]. In another study, investigating the efficacy of different antiseptic compounds in a sample of 73 Holstein heifer calves, the umbilical stump fell off at 16.3 ± 7.0 days of age; in calves dipped with 4% chlorhexidine mixed with alcohol (50:50), the umbilical stump detached at an average of 20 days compared with 15.5 days for the other three treatments (7% iodine, dry nisin, liquid nisin), but this difference was not statistically significant. No information was provided for the healing times of the umbilical wound [40].

To the authors’ knowledge, there is only one study investigating the drying times of umbilical stumps of dairy calves, whose observations, however, are limited to the first 8 days of life. Nonetheless, it demonstrated that dryness is a poor indicator of age [41].

In the dairy industry, male calves, especially pure-bred dairy calves, represent a cost in terms of feed and space required for raising them; therefore, balancing animal welfare and farmers’ interests when determining the optimum age for a calf to leave the farm of origin is a challenge.

Despite the Council Regulation (EC) No 1/2005 establishing a “completely healed navel” as an indicator of calf fitness for transport, this requirement has proven to be prone to misinterpretation and consensus among veterinary practitioners and stakeholders is currently weak.

According to our results, a completely dried and shriveled navel stump (score 3) or the presence of a scab or granulation tissue covering the umbilical wound (score 4) can be observed in calves of highly variable age (from 1 day to 2 months old), and therefore cannot guarantee that calves that are too young will not be transported.

“Navel healing” should be defined as the scarring of the umbilical wound (score 5), which occurs no earlier than 3–4 weeks of life. However, if this were the interpretation intended by the European legislator, it is clearly difficult to associate navel healing with the minimum age thresholds of 10 and 14 days established for longer journeys.

## 5. Conclusions

In conclusion, the need to address the contradiction in the European Regulation between navel condition and the minimum age at which calves can be transported for longer journeys is evident. Therefore, clarification on what is meant by “navel healing” from an anatomic-physiological point of view is required.

Alternatively, in order to reach a compromise between balancing animal welfare and farmers’ interests, good and best practice could be proposed. The presence of a scab on the umbilical wound (score 4) could represent the minimum requirement for transporting calves (except for journeys exceeding 8 h). This could be considered good practice, as the risk of transporting too young calves would be only 4.3%. Complete navel healing (score 5), since it entails 0% risk of transporting too young calves, should be considered best practice for transporting calves in general and the minimum requirement for transporting calves for journeys exceeding 8 h.

## Figures and Tables

**Table 1:** Age distribution of calves with different umbilical scores (percentage in brackets).

Umbilical Score	Age Range (Days)							Total
	0–9	10–14	15–20	21–30	31–40	41–50	51–90	
1	16 (100)	0 (0)	0 (0)	0 (0)	0 (0)	0 (0)	0 (0)	16
2	9 (75)	1 (8)	2 (17)	0 (0)	0 (0)	0 (0)	0 (0)	12
3	63 (47)	28 (21)	22 (16)	13 (10)	4 (3)	4 (3)	0 (0)	134
4	6 (9)	6 (9)	17 (26)	16 (25)	17 (26)	1 (2)	2 (3)	65
5	0 (0)	0 (0)	2 (3)	22 (31)	8 (11)	19 (26)	21 (29)	72

**Table 2:** Probability and 95% C.I. (%, in brackets) that calves with an umbilical score of at least 3 (completely dry and shriveled, inflexible umbilical stump), 4 (no umbilical stump, but scab or granulation tissue on the umbilical wound), or 5 (completely healed umbilical wound) were less than 10 days old (fit for journeys within 100 km), at least 10 days old (fit for journeys longer than 100 km), or older than 14 days (fit for journeys exceeding 8 h).

Calf Age	Umbilical Score		
	≥3	≥4	5
<10 d	25.5% (20.6–31.0)	4.3% (2.0–9.2)	0% (0–4.9)
≥10 d	74.5% (69.0–79.4)	95.7% (90.8–98.0)	100% (95.1–100)
>14 d	62.0% (56.1–67.6)	91.3% (85.4–94.9)	100% (95.1–100)

## Data Availability

The data presented in this study are openly available in Zenodo at https://doi.org/10.5281/zenodo.5776839 (accessed on 29 January 2022).

## Supplementary Materials

The following are available online at https://www.mdpi.com/article/10.3390/ani12030358/s1, Table S1: Contingency table comparing umbilical score (at least 3—completely dry and shriveled umbilical stump—or less) and calf age (at least 10 days—journeys longer than 100 km—or less), Table S2: Contingency table comparing umbilical score (at least 3—completely dry and shriveled umbilical stump—or less) and calf age (older than 14 days—journeys exceeding 8 h—or less), Table S3: Contingency table comparing umbilical score (at least 4—no umbilical stump, but scab or granulation tissue on the umbilical wound—or less) and calf age (at least 10 days—journeys longer than 100 km—or less), Table S4: Contingency table comparing umbilical score (at least 4—no umbilical stump, but scab or granulation tissue on the umbilical wound—or less) and calf age (older than 14 days—journeys exceeding 8 h—or less), Table S5: Contingency table comparing umbilical score (5—completely healed umbilical wound—or less) and calf age (at least 10 days—journeys longer than 100 km—or less), Table S6: Contingency table comparing umbilical score (5—completely healed umbilical wound—or less) and calf age (older than 14 days—journeys exceeding 8 h—or less).
