# Supplementary material for: Navel Healing and Calf Fitness for Transport
Source: Animals (Basel). 2022 Feb 1;12(3):358. doi: 10.3390/ani12030358 (PMC8833597; doi:10.3390/ani12030358)
Supplement: Supplementary file 1 [file animals-12-00358-s001.zip › animals-1542283-supplementary.pdf]

Supplementary Material

Navel Healing and Calf Fitness for Transport

Mariana Roccaro, Marilena Bolcato, Naod Thomas Masebo, Arcangelo Gentile and Angelo Peli

Table S1. Contingency table comparing umbilical score (at least 3 – completely dry and shriveled umbilical stump – or less) and calf age (at least 10 days – journeys longer than 100 km – or less).

Umbilical score	Calf age		Total
	< 10 d	≥ 10 d	
< 3	25	3	28
≥ 3	69	202	271
Total	94	205	299

Table S2. Contingency table comparing umbilical score (at least 3 – completely dry and shriveled umbilical stump– or less) and calf age (older than 14 days – journeys exceeding 8 hours – or less).

Umbilical score	Calf age		Total
	≤ 14 d	> 14 d	
< 3	26	2	28
≥ 3	103	168	271
Total	129	170	299

Table S3. Contingency table comparing umbilical score (at least 4 – no umbilical stump, but scab or granulation tissue on the umbilical wound – or less) and calf age (at least 10 days – journeys longer than 100 km – or less).

Umbilical score	Calf age		Total
	< 10 d	≥ 10 d	
< 4	88	73	161
≥ 4	6	132	138
Total	94	205	299

Table S4. Contingency table comparing umbilical score (at least 4 – no umbilical stump, but scab or granulation tissue on the umbilical wound – or less) and calf age (older than 14 days – journeys exceeding 8 hours – or less).

Umbilical score	Calf age		Total
	≤ 14 d	> 14 d	
< 4	117	44	161
≥ 4	12	126	138
Total	129	170	299

Table S5. Contingency table comparing umbilical score (5 – completely healed umbilical wound – or less) and calf age (at least 10 days – journeys longer than 100 km – or less).

**Umbilical score**	**Calf age**		**Total**
	**< 10 d**	**≥ 10 d**	
< 5	94	133	227
5	0	72	72
Total	94	205	299

Table S6. Contingency table comparing umbilical score (5 – completely healed umbilical wound– or less) and calf age (older than 14 days – journeys exceeding 8 hours – or less).

**Umbilical score**	**Calf age**		**Total**
	**≤ 14 d**	**> 14 d**	
< 5	129	98	227
5	0	72	72
Total	129	170	299
